# Transcriptional gene network involved in drought stress response: application for crop breeding in the context of climate change

**DOI:** 10.1098/rstb.2024.0236

**Published:** 2025-05-29

**Authors:** Kazuo Nakashima, Kazuko Yamaguchi-Shinozaki, Kazuo Shinozaki

**Affiliations:** ^1^ Japan International Research Center for Agricultural Sciences, Tsukuba, Ibaraki Prefecture, Japan; ^2^ Tokyo University of Agriculture, Setagaya, Tokyo, Japan; ^3^ The University of Tokyo, Bunkyo-ku, Tokyo, Japan; ^4^ Center for Sustainable Resource Science, RIKEN, Yokohama, Kanagawa, Japan; ^5^ Nagoya University, Nagoya, Aichi, Japan

**Keywords:** drought stress, gene discovery, transcriptional regulation, genetic engineering, crop breeding

## Abstract

The rapid increase in greenhouse gases has accelerated global warming, causing significant issues related to climate change, biodiversity and agriculture and adversely affecting crop production and food supply. The molecular and physiological mechanisms by which plants respond to abiotic stresses such as drought, cold and heat are well understood, according to advances in transcriptome analyses. These studies underscore the critical role of transcriptional regulation in managing drought stress and developing tolerance in *Arabidopsis* and other plants. Key genes, including those encoding transcription factors, protein kinases and other regulatory proteins, play essential roles in the cellular and molecular responses to drought. At the onset of drought stress, dehydration-induced signals relay to the nucleus, triggering the transcription of stress-related genes to cope with water deficit. Both abscisic acid (ABA)-dependent and ABA-independent regulatory mechanisms have been explored in these responses. Furthermore, many drought-inducible genes have been shown to increase stress tolerance via transgenic methods. The use of insights from *Arabidopsis* is vital for advancing crop breeding through the use of genetic modification technologies and genome editing. Recent advances in genomic technologies have provided critical data for crop genotyping, serving as essential platforms.

This article is part of the theme issue ‘Crops under stress: can we mitigate the impacts of climate change on agriculture and launch the ‘Resilience Revolution’?’.

## 1. Introduction

Carbon dioxide (CO_2_) levels have been rising throughout the 21st century. The increase in CO_2_ and other greenhouse gases has caused global warming, leading to severe problems in climate change, biodiversity and agriculture. Recently, rapid climate change has significantly impacted crop production and the supply of food [1,2]. Drought remains a critical environmental stress that negatively affects plant diversity and crop yields.

Since the 1990s, advances in plant biology have been essential for understanding plant survival strategies under severe water deficit conditions and for breeding drought-resistant crops. Research has focused on plant responses to environmental stress at the molecular level using model plants such as *Arabidopsis thaliana* [3,4]. Studies have identified numerous drought-inducible genes, revealing the molecular mechanisms of stress responses and tolerance. Systematic analyses of transcriptional regulatory networks have shed light on how plants sense, respond to and manage severe drought stress. Key aspects of transcriptional regulation include *cis*-acting elements and their transcription factors (TFs), which regulate stress-related genes and cellular responses [5–7].

This review examines the molecular mechanisms of plant responses to drought stress, especially transcriptional regulation. We aimed to elucidate the roles of drought-inducible genes in stress tolerance. This knowledge has been instrumental in developing drought-tolerant crops through advanced gene technologies. Genomic analyses have revealed new genes involved in drought tolerance, offering insights for future crop breeding.

### (a) Physiological and molecular responses to drought stress

**Figure 1.**
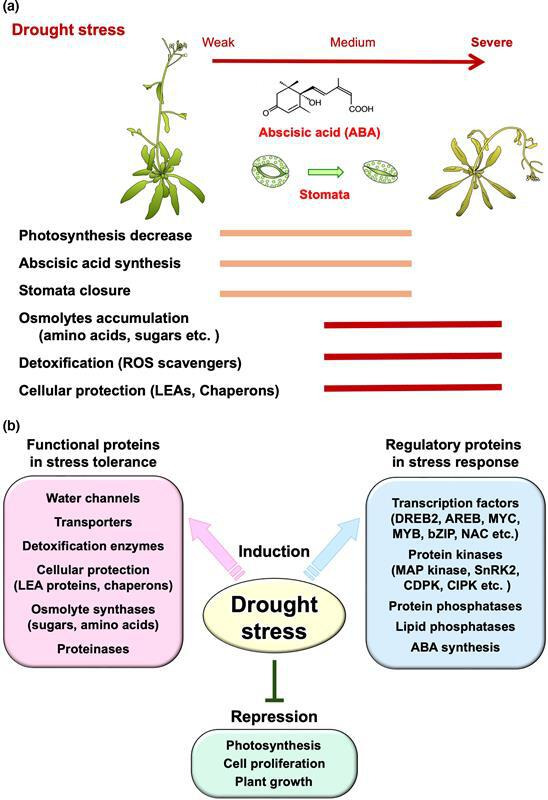
Physiological and molecular responses to drought stress. (*a*) Physiological responses to drought stress: When plants are exposed to dehydration conditions, growth and photosynthesis are inhibited. ABA is synthesized in response to drought stress and acts to close stomata, thereby reducing water loss. Various types of osmolytes, including sugars and amino acids, accumulate alongside enzymes and proteins that protect cells from dehydration. (*b*) Functions of drought-inducible gene products and their classification. Drought-inducible genes are divided into two major categories: functional proteins that contribute to stress tolerance and regulatory proteins involved in the stress response. Conversely, drought-repressed genes typically include those associated with photosynthesis and plant growth.

Land plants, which are sessile, adapt to environmental changes, including drought, cold and heat stresses, by evolving unique survival strategies [3]. Under drought conditions, water deficiency inhibits photosynthesis and growth. Abscisic acid (ABA) is synthesized, leading to stomatal closure, which reduces gas exchange and transpiration, further inhibiting photosynthesis and increasing respiration. In later drought phases, metabolites, osmolytes and stress-inducible proteins accumulate to protect cells from dehydration and oxidative stress (figure 1a).

In the 1990s, numerous genes responsive to dehydration were identified in *Arabidopsis* [8,9]. Following *Arabidopsis* genome sequencing in 2000, many drought-inducible genes were discovered via microarrays [10]. Advances in transcriptome technology have led to the identification of thousands of stress-inducible genes. Their functions have been analyzed through transgenic technology, highlighting their essential roles in stress tolerance [4]. These genes are categorized into those encoding functional proteins for stress tolerance and those encoding regulatory proteins, such as TFs and protein kinases (figure 1b). Conversely, many genes involved in photosynthesis and cell growth are repressed under drought stress, reducing growth. Most drought-inducible genes are crucial for the drought response and tolerance, which will be further discussed below. Regulatory genes will be addressed in the next section.

### (b) Drought-inducible genes for abscisic acid biosynthesis, metabolism and transport

ABA, a crucial phytohormone, influences various plant life stages, from seed development to adult stress responses. It regulates stomatal closure and stress-responsive gene expression and is controlled by biosynthesis, catabolism and modification levels [11]. The key genes involved in ABA biosynthesis include the 9-*cis*-epoxycarotenoid dioxygenase (NCED) enzymes identified in *Arabidopsis*. NCED3, in particular, is vital because it is strongly induced under dehydration and essential for maintaining ABA levels for effective stomatal response to drought [12]. Conversely, cytochrome P450 monooxygenase family A (CYP707As), and in particular CYP707A2, play a significant role in ABA degradation during rehydration [13,14]. The regulation of ABA involves its synthesis primarily in leaf vascular tissues and its transport to guard cells for stomatal closure [15]. ABA transport involves multiple transporter types, including ATP-binding cassette (ABC) family transporters, nitrate transporters (NPFs) and detoxification efflux carrier (DTX) family transporters [16]. ABCG25 exports ABA in xylem tissues, whereas ABCG40 in guard cells imports it, aiding in stomatal closure [17,18]. NPF4.6 and NPF5.1 transport ABA from vascular to guard cells [19,20], and DTX50, which is expressed in the leaf vasculature [21], also supports stomatal closure. These mechanisms collectively ensure that ABA is effectively transported and regulated in response to water deficit [16].

### (c) Drought-induced genes for transporters and channels

Transporters play vital roles in maintaining the water status of plant tissue for drought tolerance. A key group is the aquaporin family, in which major water channel proteins belong to the major intrinsic protein (MIP) group [22]. These proteins form pores in cell membranes, with tonoplast intrinsic proteins (TIPs) in vascular membranes and plasma intrinsic proteins (PIPs) in plasma membranes, facilitating intra- and transcellular water transport. For example, the PIP gene *RD28* in *Arabidopsis* is significantly upregulated by dehydration, which is crucial for cellular water homeostasis [8].

Ion channel proteins, which are critical in stomata and other tissues during drought, regulate various functions [23]. Increases in cytoplasmic Ca^2+^ activate two types of anion channels: slow-activating sustained (S-type) and rapid-transient (R-type) channels, which induce membrane depolarization [24]. This process inhibits inwards K^+^ channels (KAT1/KAT2), controlling stomatal closure. K^+^ channels, including K^+^ uptake transporters (KUPs) and guard cell outwards rectifying K^+^ channels (GORKs), maintain osmotic homeostasis and cell turgor [25]. Mutations in these channels reduce the stomatal response and survival under dehydration.

### (d) Drought-inducible genes involved in stress tolerance

Proteins crucial for the biosynthesis and transport of osmolytes, such as sugars and amino acids, accumulate significantly during dehydration. Carbohydrates such as raffinose and galactinol from the raffinose family of oligosaccharides (RFOs) and tricarboxylic acid cycle products (citrate, malate and succinate), as well as monosaccharides (glucose and fructose), accumulate in response to dehydration [26]. Galactinol synthase genes (*GolS*) encode key enzymes for the production of oligosaccharides in the raffinose family, especially *GolS2*, whose expression is strongly induced under drought stress, and GolS2 overexpression increases drought tolerance in transgenic plants [27].

Among amino acids, branched-chain amino acids (valine, leucine, isoleucine), aromatic amino acids (tyrosine, tryptophan) and members of the glutamate family (glutamate, histidine), including proline, increase during dehydration [26]. The *BCAT2*, *LKR/SDH*, *P5CS* and *ADC2* genes, which facilitate the synthesis of these amino acids, exhibit dehydration-inducible expression. Specifically, the *P5CS* gene is crucial for proline accumulation, whereas the *ProDH* gene regulates proline catabolism upon rehydration [28,29]. Additionally, plant secondary metabolites play essential roles in stress response and tolerance [30].

Under drought stress, reactive oxygen species (ROS) accumulate due to reduced CO_2_ levels and excess energy, leading to stress-dependent ROS production across different organelles. The expression of genes encoding ROS-scavenging enzymes such as superoxide dismutase (SOD), ascorbate peroxidase (APX), glutathione peroxidase (GPX) and catalase is induced, helping mitigate ROS overproduction [31]. The overexpression of these genes in transgenic plants enhances drought tolerance.

Under severe drought, late embryogenesis abundant (LEA) proteins increase to protect cells from dehydration. In *Arabidopsis*, drought-inducible *RD* and *ERD* genes, along with cold-inducible *COR* genes, encode various LEA proteins. These proteins are hydrophilic and intrinsically disordered, shielding enzymes, structural proteins and membranes under dehydration [32]. The overexpression of *LEA* genes in transgenic plants increases drought tolerance. Other critical proteins, such as heat shock proteins (HSPs), accumulate in response to drought and heat. HSPs prevent aggregation and assist in refolding denatured proteins, which are categorized into groups such as HSP40, HSP60, HSP70, HSP90 and HSP100, with HSP60 and HSP70 functioning as chaperonins [33].

## 2. Mechanism of gene expression under drought stress

**Figure 2.**
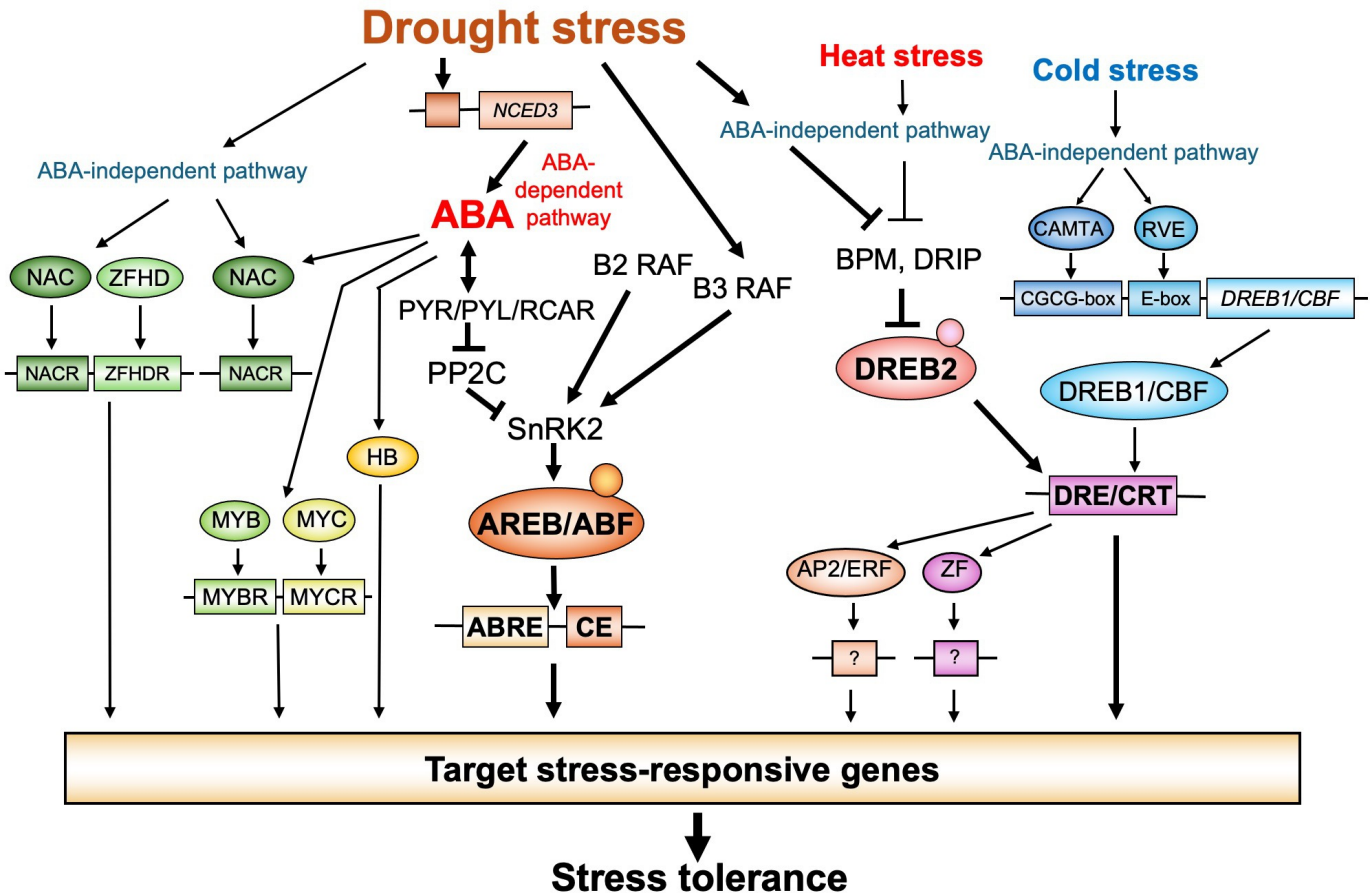
Transcriptional regulatory networks under drought, cold and heat stress conditions. The coloured ellipses represent transcription factors (TFs) that control stress-inducible gene expression. Boxes indicate *cis*-acting elements in promoters involved in stress-responsive gene expression. The small filled circles denote modifications of TFs in response to stress signals, such as phosphorylation. Abbreviations include ABA receptors (PYR/PYL/RCAR), protein kinases (SnRK2, B2-RAF, and B3-RAF), protein phosphatases (PP2C) and E3 ligases (BPM and DRIP). The regulatory cascade of stress-responsive gene expression is illustrated from top to bottom. The thick black arrows highlight the major signalling pathways that regulate many downstream genes.

The regulatory mechanisms of gene expression under drought stress have been extensively studied in *Arabidopsis*. Transcriptome analyses of genes induced by drought stress via ABA-deficient (*aba*) or ABA-insensitive (*abi*) mutants revealed that drought-responsive gene expression is regulated by both ABA-dependent and ABA-independent pathways. Extensive research on both pathways has identified many TFs involved in each pathway [3,34] (figure 2).

### (a) AREB/ABF transcription factors in abscisic acid-dependent gene expression

In ABA-dependent transcription, the ABRE (ABA-responsive element: ACGTGG/TC) is a key *cis*-acting element in promoters that regulate ABA-responsive gene expression. A single ABRE is insufficient; multiple ABREs or an ABRE with a coupling element (CE) are needed. CE1, CE3, motif III and DRE/CRT have been identified as CEs, many of which share similarities with ABREs and contain an A/GCGT motif [35].

Basic-domain leucine zipper (bZIP) TFs, known as ABRE-binding (AREB) proteins or ABRE-binding factors (ABFs), bind to ABRE sequences and act as transcriptional activators in ABA-dependent gene expression [36,37]. *Arabidopsis* has 78 bZIP family members, which are grouped into 13 categories on the basis of sequence similarity [38]. AREB/ABFs belong to the Group A subfamily, which consists of nine homologues in *Arabidopsis*. These proteins contain three conserved N-terminal domains (C1, C2 and C3) and one C-terminal domain (C4). Among them, the *AREB1/ABF2*, *AREB2/ABF4, ABF3* and *ABF1* genes are expressed in vegetative tissues, and their expression is upregulated by ABA and osmotic stress. Genetic analysis of multiple *areb/abf* mutant plants has shown that these four AREB/ABF proteins are functionally redundant and collectively regulate a major portion of ABA-responsive genes [39] (figure 2).

AREB/ABFs require ABA-dependent phosphorylation at multiple conserved sites for activation. SUCROSE NON-FERMENTING1-RELATED PROTEIN KINASE2s (SnRK2s) phosphorylate these sites in the presence of ABA [40]. *Arabidopsis* encodes 10 SnRK2 family proteins, which are classified into three subclasses. At least nine genes are activated by osmotic stress [41,42]. Among these nine genes, three SnRK2s—SRK2D/SnRK2.2, SRK2I/SnRK2.3 and SRK2E/SnRK2.6/OPEN STOMATA1 (OST1)—belong to subclass III, which is strongly activated by ABA. These SnRK2s phosphorylate targets, including AREB/ABFs, converting them into their active forms and inducing ABA-responsive gene expression [43–45].

The largest *Arabidopsis* protein phosphatase family, protein phosphatase 2C (PP2C), includes 76 members [46]. Nine belong to clade A and are critical negative regulators of ABA signalling, including ABA INSENSITIVE 1 (ABI1) and ABI2 [47]. In the absence of ABA, ABI1 interacts with subclass III SnRK2s, dephosphorylating and inhibiting them [48]. Six clade A PP2Cs—ABI1, ABI2, HYPERSENSITIVE TO ABA1 (HAB1), HAB2, ABA HYPERSENSITIVE GERMINATION 1 (AHG1) and AHG3—target and dephosphorylate SnRK2s. HAB1 and HAB2 function similarly to ABI1 [49,50], whereas AHG1 and AHG3 are involved in seed development and dormancy. The remaining clade A PP2C members included three homologues: HIGHLY ABA-INDUCED 1 (HAI1), HAI2 and HAI3. These genes are ABA inducible, and *hai* triple mutants are hypersensitive to ABA, suggesting that HAIs may play a role in the negative feedback regulation of ABA signalling, although their specific targets remain unclear [44,51].

The PYRABACTIN RESISTANCE1/PYR1 LIKE/REGULATORY COMPONENTS OF ABA RECEPTOR (PYR/PYL/RCAR) family of START proteins, which includes 12 functionally redundant members, are soluble ABA receptors crucial for activating subclass III SnRK2s [52,53]. Under drought stress, ABA accumulates, binds to PYR/PYL/RCARs and inhibits PP2C activity, releasing SnRK2s from inhibition. SnRK2s were initially believed to self-activate via autophosphorylation, but they are now understood to be activated by Group B MAP KINASE KINASE KINASE (MAPKKK) Raf-like kinases: B2 Raf-like kinases (B2-RAFs: RAF7, RAF10, RAF11 and RAF12) and B3 Raf-like kinases (B3-RAFs: RAF3/M3Kδ1, RAF4/M3Kδ7, RAF5/M3Kδ6 and RAF6/M3Kδ5) under osmotic stress [54–56]. B2-RAFs exhibit constitutive kinase activity, whereas B3-RAFs are specifically activated under osmotic stress conditions [57]. Thus, autophosphorylation of subclass III SnRK2s is insufficient for ABA responses; B2-RAFs are essential for subclass III SnRK2 activation in response to ABA, whereas B3-RAFs increase subclass III SnRK2 activity under drought conditions. The activated SnRK2s subsequently turn on AREB/ABF TFs, which activate ABA-responsive gene expression in an ABRE-dependent manner under drought stress [5,57].

### (b) DREB2 transcription factors in abscisic acid-independent gene expression

In the ABA-independent transcription system under drought stress, a dehydration-responsive element (DRE) with the core sequence A/GCCGAC has been identified as a *cis*-acting element that regulates the expression of dehydration- and cold-inducible genes in *Arabidopsis* [58]. DREs have also been detected in the promoter regions of many dehydration- and cold-inducible genes in various plant species [3]. A similar element, the C-repeat motif (CRT), has been identified in the promoter regions of cold- and drought-inducible genes known as cold-regulated (*COR*) genes [59]. The *Arabidopsis* TFs DREB1A/CBF3 and DREB2A specifically bind to DRE/CRT [60]. *DREB1A/CBF3* expression is induced by cold stress, whereas *DREB2A* expression is induced by drought, high-salt and heat stresses [60,61]. Both proteins bind to DRE/CRT, with DREB1A/CBF3 being involved in cold-responsive gene expression and DREB2A being involved in drought-responsive gene expression (figure 2).

The AP2/ERF family is a large group of plant-specific TFs, with 145 members in *Arabidopsis* classified into four major subfamilies: AP2, RAV, ERF and DREB [62]. The DREB subfamily is further divided into six subgroups: A-1 to A-6. DREB1A/CBF3 belongs to A-1, and DREB2A belongs to A-2. The A-1 (DREB1/CBF) subgroup includes six members [62], among which DREB1A/CBF3, DREB1B/CBF1 and DREB1C/CBF2 are rapidly induced by cold stress. The importance of these three cold-inducible DREB1/CBFs in cold stress responses has been clearly demonstrated [63]. The DREB2 subgroup comprises eight members (DREB2A−2H), with DREB2A and DREB2B functioning mainly in terms of gene expression in response to drought, high salt and heat stress [64]. DREB2A and DREB2B are key TFs involved in dehydration- and heat-inducible gene expression via DRE/CRT in an ABA-independent manner [6,65]. Additionally, DREB1s and DREB2s regulate many target genes, including other AP2/ERF-type and ZF-type TFs, to form transcriptional cascades.

In *Arabidopsis*, the overexpression of *DREB1/CBF* genes increases the expression of many target stress-inducible genes and improves stress tolerance to dehydration and freezing [60,66]. In contrast, overexpression of *DREB2A* neither induces the expression of target genes nor improves stress tolerance, suggesting that DREB2A requires posttranslational modifications, such as phosphorylation, for its activation [6]. DREB2A has a negative regulatory domain (NRD) behind the DNA-binding domain, and deleting NRD converts DREB2A into a constitutively active form (DREB2A CA). The NRD consists of approximately 30 amino acid residues rich in serine and threonine and is predicted to be a PEST sequence, which functions as a degradation signal conserved among eukaryotes. Transgenic plants constitutively expressing *DREB2A CA* presented improved stress tolerance to drought and heat. Many dehydration-responsive genes, such as LEA proteins and heat-shock protein genes, are induced in these transgenic plants [65]. The partial reduction in dehydration- or heat-shock-responsive gene expression in *dreb2a* mutants indicates that DREB2A plays important roles in gene expression in response to dehydration and heat-shock stress [65]. In *Arabidopsis*, overexpression of *DREB1A/CBF3* improves tolerance to dehydration and freezing, whereas overexpression of *DREB2A CA* enhances tolerance to dehydration and heat shock but not to freezing. Although DREB1A/CBF3 and DREB2A CA have overlapping target genes, some are more specific to each DREB: carbohydrate metabolism enzyme genes have greater specificity for DREB1A/CBF3, and molecular chaperone genes have greater specificity for DREB2A CA. Promoter analyses revealed that DREB1A/CBF3 prefers A/GCCGACNT, whereas DREB2A prefers ACCGAC, contributing to differences in target genes [61,67].

DREB2A is highly unstable because the NRD is located in the centre of the protein [62]. BTB/POZ AND MATH DOMAIN proteins (BPMs), which are substrate adaptors for Cullin3 (CUL3)-based E3 ligases, interact with DREB2A via the NRD, leading to its degradation. In BPM-knockdown plants, DREB2A accumulates and increases the expression of its target genes under stress [68]. NRD is highly phosphorylated under normal conditions, likely via casein kinase 1, and its phosphorylation decreases during stress conditions, promoting DREB2A degradation by enhancing its interaction with BPM2 [69]. Additionally, SUMOylation of a lysine residue near the NRD during heat stress inhibits this interaction [70]. DREB2A is also regulated by the E3 ligases DREB2A-INTERACTING PROTEIN1 (DRIP1) and DRIP2, which mediate its ubiquitination and degradation. In DRIP1 and DRIP2 knockouts, DREB2A is stabilized, leading to increased expression of its target genes [71].

RADICAL-INDUCED CELL DEATH 1 (RCD1) is another candidate involved in DREB2A degradation. RCD1 interacts with the RCD1-interacting motif (RIM) in the C-terminus of DREB2A [72], potentially mediating its degradation. NUCLEAR FACTOR Y SUBUNIT C10 (NF-YC10), also known as DNA POLYMERASE II SUBUNIT B3−1 (DPB3−1), was identified as a protein that interacts with DREB2A [73]. NF-YC10 forms a trimer with NF-YA2 and NF-YB3, which have both positive and negative effects on heat- and drought-inducible DREB2A target genes, respectively. In contrast, NF-YB2 positively affects drought-inducible DREB2A target genes [74]. MEDIATOR 25 (MED25) negatively regulates DREB2A by altering its conformation and inhibiting DNA binding [75].

### (c) Other transcription factors involved in drought stress responses

In response to drought, plants utilize various TFs beyond DREB/CBFs and AREB/ABFs to regulate gene expression precisely, ensuring survival and growth in changing environments. Among them, NAC (NAM, ATAF1/2 and CUC) family proteins are plant-specific TFs, with more than 100 *NAC* genes identified in *Arabidopsis* and rice, contributing to both development and abiotic stress responses [76]. Stress-responsive NACs (SNACs), such as ANAC019, ANAC055 and ANAC072/RD26, regulate many drought-responsive genes and promote ABA-responsive leaf senescence [77–79]. These NACs also cooperate with the ZF-HD TF ZFHD1 to regulate drought-inducible gene expression. They bind to specific sequences such as ‘CACG’ for the NAC recognition sequence (NACRS) and ‘TA/TAATTNNC’ for the ZFHD recognition sequence (ZFHDRS) [80]. Another NAC, ANAC096, functions in concert with AREB/ABF in ABA signalling during drought [81].

The drought-inducible *RD22* gene is regulated by ABA but not by AREB/ABFs. Its expression is controlled by MYC and MYB recognition sites in the RD22 promoter. The overexpression of the MYC2 and MYB2 TFs in plants enhances ABA sensitivity and osmotic stress tolerance [82,83]. Many genes, including ABA-inducible and jasmonic acid (JA)-inducible genes, are upregulated in transgenic plants. Conversely, MYC2 mutants present reduced ABA sensitivity and lower expression of ABA- and JA-inducible genes. Thus, MYC2 is also a key regulator of JA-responsive gene expression in plants and acts as a negative regulator of blue-light-mediated photomorphogenic growth [84]. MYC2 likely integrates the ABA, JA and light signalling pathways in *Arabidopsis*.

The WRKY family is another major group of plant TFs, with 74 members in *Arabidopsis* and 109 in rice. WRKYs regulate various plant responses, including those to biotic and abiotic stresses, by interacting with W-box (TTGACC/T) sequences in promoter regions [85]. Several WRKY TFs are involved in drought responses. For example, *Arabidopsis* WRKY63 enhances the expression of *AREB1/ABF2*, promoting ABA-responsive expression [86].

The Cys2/His2-type zinc-finger proteins AZF1, AZF2, AZF3 and STZ/ZAT10 act as transcriptional repressors by binding to A(G/C)T repeats. The expression of these genes is induced by drought, high salt and cold stresses, and they contribute to stress tolerance [87,88]. AZF1 and AZF2, in particular, repress genes downregulated by osmotic stress and ABA, including many auxin-responsive genes, such as *small auxin-up RNA* (*SAUR*) genes, and may regulate growth under stress conditions [89]. Homeobox 6 (HB6), a class I homeodomain-leucine zipper (HD-Zip) TF, is induced by drought and ABA and negatively regulates ABA-mediated drought responses. Through its interaction with ABI1, a key regulator of ABA responses, HB6 may act as a master switch for ABA-specific adaptations [90]. Two *Arabidopsis* TFs have been identified with specific roles in drought stress responses: CIN-like TCP13, which regulates plant growth [91] and DREB26/ERF12, which is involved in cuticular wax biosynthesis [92]. TFs in various crops that contribute to drought response have been comprehensively reviewed [93]. Many TFs coordinate plant responses to drought by controlling complex gene expression networks and interacting with pathways related to biological stress, light, hormones and growth, helping plants adapt and continue growing in ever-changing environments.

## 3. Breeding of crops resilient to drought stress

**Figure 3.**
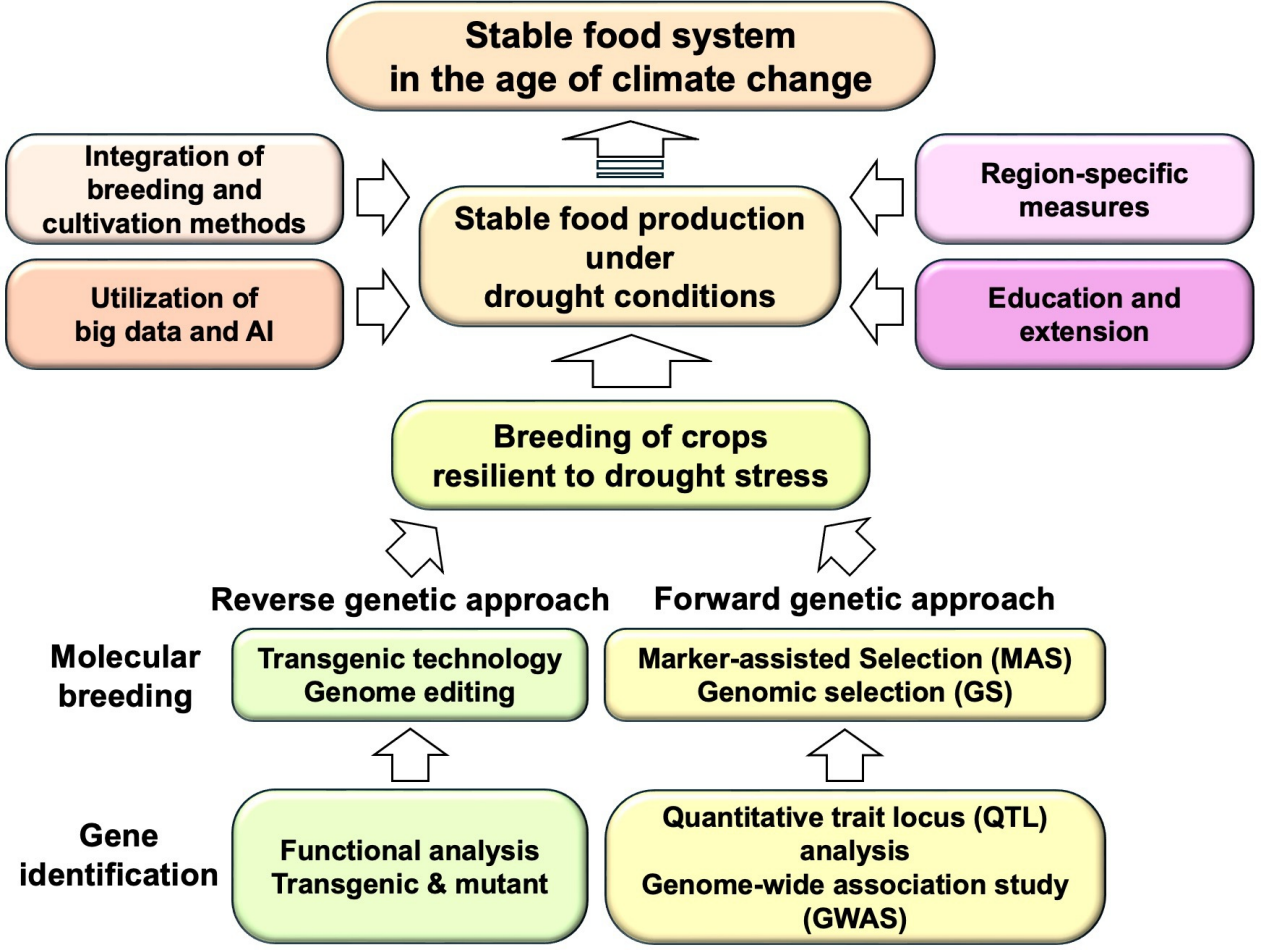
Schematic diagram of the breeding of crops resilient to drought stress and related technologies to achieve a stable food system under climate change. Key genes that contribute to increasing drought tolerance can be identified and utilized through both forward genetic and reverse genetic approaches. Integration of breeding and cultivation methods, utilization of big data and AI, region-specific measures, and education and extension are also expected to contribute to stable food production under drought conditions.

The development of drought-resistant crops is an important issue in dealing with the problems of climate change and shortage of water resources. In fact, research is underway on four major breeding crops—wheat, rice, maize and soybean—that are particularly resistant to climate change. As mentioned previously, great progress has been made to understand the molecular mechanisms behind climate change and drought tolerance, mechanisms that use reverse genetic approaches and that are put to potential use by providing information and candidate molecules in breeding. Moreover, by means of germplasm collection and genotypic and phenotypic analyses, not only drought-tolerant genes and alleles are identified, but they are also utilized in breeding by way of forward genetic approaches [6]. In this section, we will discuss two main approaches that enhance crop tolerance to drought (figure 3).

### (a) Reverse genetic approach

As indicated above, many drought-response and/or tolerance-related genes, including TFs, have been identified. With the help of biotechnology, transgenic crops containing these genes are developed, and their effects on drought tolerance are studied in controlled environments such as laboratories, greenhouses, and in part, in field tests. Regulatory protein genes involved in transcription and signal transduction have been examined for their potential use to enhance drought tolerance in crops. For example, TFs such as DREB1/CBF and its homologues (which control the expression of many genes involved in environmental stress tolerance) have been used to increase drought tolerance in various crop species, including rice; some do well in field evaluations [5,94–96]. Furthermore, sugarcane overexpressing DREB2A CA, soybean overexpressing AREB1 and rice overexpressing NAC-type TFs demonstrate superior performance in the field [97–99]. Because some TFs can cause growth inhibition, it is more effective to use an appropriate promoter, such as a stress-inducible promoter, and then to control the timing of expression. When the CCCH tandem zinc finger protein-encoding gene *OsTZF5* (putatively involved in RNA binding and stability) is overexpressed under a stress-inducible promoter, it reduces the impact of drought stress on rice grain yield under field conditions [100]. Mega *et al*. [101] show that overexpression of the wheat ABA receptor TaPYL enhances ABA sensitivity in wheat, thereby reducing water consumption significantly. Physiological analysis demonstrates that this water-saving trait is the result of reduced transpiration and associated increased photosynthetic activity, both of which increase grain yield per litre of water and protect productivity during water shortage.

Functional protein genes involved in metabolism and other areas are also utilized to increase drought tolerance in crops. By overexpressing the *OsLEA3−1* gene under an appropriate promoter, Xiao *et al*. [102] generate transgenic rice with a significantly increased tolerance to drought and no yield loss. The introduction of *Arabidopsis GolS2* into rice increases the levels of oligosaccharides (e.g. osmoprotectant galactinol) and maintains yield in the field under drought [103].

In this way, various studies have demonstrated that drought tolerance can be improved with appropriate promoters by overexpressing important factors, many of which are conserved and can be utilized across species. The same function is observed even when such factors from different species are introduced, indicating that many land plants have mechanisms conserved for drought tolerance in the course of evolution. On the other hand, it should be emphasized that precautions should be taken in applying the results of model plants to crops. In model plants, drought tolerance is evaluated during the vegetative growth stage under controlled conditions, whereas in crops, it is done on parts harvested under complex environments. Previous research in crop science shows that drought has the greatest impact on yield during the flowering stage, which is the appropriate time to apply drought stress in order to evaluate yield.

To date, several drought-tolerant transgenic crops have been approved and commercialized. In maize, the *cspB* gene encoding the RNA chaperone cold-shock protein B from *Bacillus subtilis* is used to confer resistance to drought [104]. The homeodomain-leucine zipper TF gene *HaHB4* from sunflower improves drought tolerance in wheat [105]. As in Gupta [106], the drought-tolerant transgenic wheat HB4® has been approved for sale and consumption as food and/or feed in more than 10 countries as well as for commercial cultivation in Argentina and Brazil. Likewise, drought-tolerant soybeans are approved for commercial production in Brazil and for food use in Australia and New Zealand [107].

Besides GM technology, genome editing has emerged as an innovative method that can be applied using reverse genetic strategies. The clustered regularly interspaced short palindromic repeats/CRISPR-associated proteins (CRISPR-Cas9) system is used extensively. With improved performance under drought conditions, mutant crops have already been created using CRISPR-Cas9. In maize, editing the promoter of the gene encoding the negative regulator of the ethylene response, ARGOS8, increases grain yield under drought conditions in the field [108]. Recently, Karavolias *et al*. [109] are able to moderately reduce stomatal density in rice by genome-editing the paralog of STOMAGEN—a positive regulator of stomatal development, EPIDERMAL PATTERNING FACTOR-LIKE10 (EPFL10)—thereby, providing a climate-adapted approach to protect rice yield. Although genome-edited crops designed to increase abiotic stress tolerance have not been commercialized yet, such crops may soon emerge in the market in the form of genome-edited varieties of nutrition-improved tomatoes [110,111].

### (b) Forward genetic approach

Forward genetic approaches are able not only to identify new genes and alleles which improve drought tolerance in genetic resources, but also to show important mechanisms that determine performance under drought stress conditions. With respect to deep rooting for improved drought avoidance, Chen *et al*. [112] have identified, through quantitative trait locus (QTL) mapping, root length-related loci that contribute to improving the root system using recombinant inbred lines derived from long-rooted varieties, which are discovered by evaluating soybean genetic resources. Uga *et al*. [113] have detected, also by QTL mapping, in an upland rice variety the *DEEPER ROOTING1* (*DRO1*) gene that causes crown roots to grow vertically. Near-isogenic lines with the *DRO1* allele of Kinandang Patong in the IR 64 genetic background show deeper roots and higher grain yields than IR 64 in drought-stricken fields. The maize *DRO1* homolog is found to be associated with differences in root angle and in drought avoidance between modern maize and the ancient species *Z. luxurians*, therefore suggesting that the function of *DRO1* is conserved among plants [114]. Another gene associated with root phenotypes has been identified by way of genome-wide association study (GWAS) [115]. This gene, named *DROUGHT1* (*DROT1*), improves drought tolerance by regulating cell wall structure, and SNPs in the promoter are responsible for the difference in transcription levels. Additionally, in maize, a GWAS of approximately 370 inbred lines identifies key genes related to drought tolerance in maize seedlings [116,117]. One of these genes, *ZmNAC111*, encodes a NAC-type TF, and its expression is reduced when a transposon is inserted into the promoter of *ZmNAC111*. Polymorphisms in the promoter of *ZmDREB2.7*, encoding a DREB2-type TF, are also found to be linked to drought tolerance [118]. Genome resequencing and SNP and structural variant analysis of the drought-tolerant maize line CIMBL55 show that the line contains 65 of 108 previously identified drought tolerance candidate alleles, including those encoding two drought-related TFs ZmABF4 and ZmNAC075 [117,119,120]. Mei *et al*. [121] report a *DREB* gene (*TaDTG6-B*) by GWAS that is closely associated with drought tolerance in wheat. These findings indicate that crop plants use multiple mechanisms to cope with drought, including drought avoidance, such as root length, and drought tolerance, including mechanisms controlled by TFs such as DREB, and that the key factors identified through reverse genetic approaches are also supported by forward genetic approaches.

Molecular breeding methods have advanced such that they are now able to accelerate candidate selection by crossing and genomic information. The useful loci cited above can be used for breeding by means of marker-assisted selection (MAS) [122–124]. In addition, recent advances in high-throughput genotyping, phenotyping and computational modelling make possible the prediction of traits in each progeny through the combination of polymorphic markers and large-scale phenotypic data—a method called genomic selection (GS) that is widely used to develop cultivated drought-tolerant crops [125–127].

## 4. Future perspectives

Recent advances in genomics and transcriptome analysis technology underscore the importance of molecular mechanisms in plant drought resistance, transcriptional regulation in particular. It is now possible to increase drought resistance in crops by modifying the expression of TFs and crucial functional proteins that contribute to stress resistance. As a result, the application of genetically modified crops has progressed significantly. On the other hand, while genome editing exhibits promise, further research in selecting useful genes is needed. Similarly, hopes are high that smart breeding utilizing genetic resources and genomic information will facilitate the creation of climate-tolerant crops. In this connection, we believe that the following comprehensive initiatives are just as important now as well as in the future (figure 3):

### (a) Integration of breeding and cultivation methods

Combining climate-tolerant crops with proper cultivation methods should provide the greatest benefits. These methods include, among others, appropriate sowing time and variety selection, strengthening root systems, soil improvement and organic matter application, conservation tillage, mulching, precision agriculture, use of plant growth regulators and inoculation with beneficial microorganisms. As for the latter two, ABA and its agonists, small molecule peptides, acetic acid and ethanol are likely to be used as plant growth regulators to increase drought tolerance [128–132]; among the rhizosphere bacteria, there are beneficial species called ‘Plant Growth-Promoting Rhizobacteria’ (PGPR) that benefit the host plant to overcome and to survive the effects of drought stress by promoting various direct and indirect responses [133]. However, the capabilities of PGPR and the needs of crops vary widely and are influenced by the environment and surrounding microorganisms; thus, more research is needed to elucidate the complexities of the interactions between different PGPR and plant species or the environment.

### (b) Utilization of big data and AI

It is said that the use of big data analysis and AI will allow us to gain new insights into drought stress tolerance. It is also anticipated that the identification of promoter regions of key genes and the effective modification of complex gene expression networks are going to be facilitated with the use of AI and mathematical models. Additionally, because actual droughts are complex, especially when they occur under long-term stress with no visible damage and under combined stress of drought and intense heat, gene expression and epigenetic regulation remain challenging. As of late, a comprehensive analysis of field soybeans in an experimental system using ridges shows that the amount of phosphate in the plant decreases before the ABA response occurs in the early stages of drought, thereby resulting in a phosphate deficiency response [134]. Moreover, the development of technology to monitor soil moisture and weather data in real-time using sensors and IoT technology is progressing so much so that it is expected to speed up the evaluation of breeding materials. Besides, precision or smart agriculture in optimal irrigation schedules and fertilization planning is now possible. Although it has already been put into practical use in developed countries, it is forecast that such technology is likely to become available in the near future for use in developing regions where droughts occur frequently.

### (c) Region-specific measures

For both breeding and cultivation methods, customized strategies are needed to suit the climatic conditions and agricultural environment of individual regions. In other words, the interaction among G (genes) × E (environment) × M (management) ought to be considered. For instance, a genotype of crop that is shown to grow well in one place will not necessarily do as well or better in another. Hence, it is recommended that useful genes be introduced into varieties and appropriate cultivation management be implemented in a way that is suitable to a specific region. Since climate change is accelerating, breeding goals must be set and take into account the unique environment and particular circumstances of each region 10 to 20 years from now.

### (d) Education and extension

It is not easy to get farmers to understand the usefulness and the dissemination of new varieties of crops developed by new breeding technologies. They might have reservations about adopting new cultivation technologies that had not been used before. It is essential to promote practical application and dissemination by way of technical guidance and information sharing with farmers and relevant parties.

The foregoing R&D and dissemination efforts play an important role to realize sustainable agriculture and food security. The key to success is to utilize the latest scientific knowledge and to adopt a multidisciplinary approach whereby researchers from various fields work together.

## Data Availability

This article has no additional data.
